# Supplementary material for: Associations of the CYP7A1 Gene Polymorphisms Located in the Promoter and Enhancer Regions with the Risk of Acute Coronary Syndrome, Plasma Cholesterol, and the Incidence of Diabetes
Source: Biomedicines. 2024 Mar 9;12(3):617. doi: 10.3390/biomedicines12030617 (PMC10968401; doi:10.3390/biomedicines12030617)
Supplement: Supplementary file 1 [file biomedicines-12-00617-s001.zip › Supplementary Table S1.pdf]

Supplementary Table S1. Allele and genotype frequencies of *CYP7A1* gene polymorphisms in ACS patients and healthy controls

Polymorphic Site (rsID-number)	ACS n=1317 (n[%])	Controls n=1046 (n[%])	*p
rs2081687 *C/T*			
Allele			
C	2163 (82.1)	1785 (85.4)	0.001
T	471 (17.8)	305 (14.6)	
Genotype			
CC	898 (68.2)	758 (72.5)	
CT	367 (27.9)	269 (25.7)	
TT	52 (4)	18 (1.7)	0.001
rs9297994 *G/A*			
Allele			
A	2166 (82.2)	1796 (85.9)	0.0005
G	468 (17.8)	294 (14.0)	
Genotype			
AA	900 (68.3)	766 (73.3)	
AG	366 (27.8)	264 (25.3)	
GG	51 (3.9)	15 (1.4)	0.0003
rs10107182 *C/T*			
Allele			
T	2216 (84.1)	1777 (85.9)	0.043
C	418 (15.9)	291 (14.0)	
Genotype			
TT	940 (71.4)	758 (73.3)	
TC	336 (25.5)	261 (25.2)	
CC	41 (3.1)	15 (1.4)	0.008
rs10504255 *A/G*			
Allele			
A	2193 (83.2)	1798 (86.1)	0.005
G	441 (16.7)	288 (13.8)	
Genotype			
AA	920 (69.9)	772 (74.0)	
AG	353 (26.8)	254 (24.4)	
GG	44 (3.3)	17 (1.6)	0.009
rs1457043 *C/T*			
Allele			
T	1982 (75.2)	1559 (74.8)	
C	652 (24.7)	523 (25.0)	NS
Genotype			
TT	756 (57.4)	579 (55.6)	
TC	470 (35.7)	401 (38.5)	NS
CC	91 (6.9)	61 (5.9)	
rs8192870 *G/T*			
Allele			
G	2138 (81.1)	1760 (84.2)	
T	496 (18.8)	328 (15.7)	0.005
Genotype			
GG	878 (66.7)	736 (70.5)	
GT	382 (39.0)	288 (27.6)	
TT	57 (4.3)	20 (1.9)	0.0001
rs3808607 *G/T*			
Allele			
T	1993 (75.6)	1573 (75.4)	
G	641 (24.3)	511 (24.5)	NS
Genotype			

TT	761 (57.8)	588 (56.4)	
GT	471 (35.8)	397 (38.1)	
GG	85 (6.5)	57 (5.5)	NS

Data are shown as n and frequency. *chi-square test. NS: No significant
