# Supplementary material for: Associations of the CYP7A1 Gene Polymorphisms Located in the Promoter and Enhancer Regions with the Risk of Acute Coronary Syndrome, Plasma Cholesterol, and the Incidence of Diabetes
Source: Biomedicines. 2024 Mar 9;12(3):617. doi: 10.3390/biomedicines12030617 (PMC10968401; doi:10.3390/biomedicines12030617)
Supplement: Supplementary file 1 [file biomedicines-12-00617-s001.zip › Supplementary Table S2.pdf]

Table Supplementary S2. Distribution of plasma lipids concentration according to the different genotypes of the rs2081687 *T/C,* rs8192870 *G/T,* rs9297994 *G/A*, rs10107182 *C/T* and rs10504255 *A/G* polymorphisms in healthy control group.

CYP7A1	*rs2081687 T/C*			
Genotypes	*CC (n=758)*	*CT (n=269)*	*TT (n=18)*	*p-value**
Parameters				
BMI (kg/m^2)	27.8 [25.5-31]	28.2 [25.4-31]	26.6 [24-31]	0.278
Blood pressure (mmHg)				
Systolic	111 [103-122.5]	112.5 [104-122]	114 [103-120]	0.954
Diastolic	70 [64.5-76]	71 [65.5-76]	71.8 [68-78.6]	0.294
Glucose (mg/dl)	90 [84-98]	89 [84-96]	85 [81-95]	0.378
Total cholesterol (mg/dl)	189.2 [166-210]	191 [169-211.5]	190 [180-223]	0.330
HDL-C (mg/dl)	45 [36.4-55.5]	45 [35.8-53.4]	46 [38.6-51]	0.583
LDL-C (mg/dl)	114.5 [95.4-132]	117.3 [95.5-136.5]	119 [108.4-183]	0.365
Triglycerides (mg/dl)	142.5 [108-202]	156.6 [103-203]	122.3 [101.5-183]	0.957
CYP7A1	*rs9297944 G/A*			
Genotypes	*AA (n=766)*	*AG (n=264)*	*GG (n=15)*	*p-value*
Parameters				
BMI (kg/m^2)	27.9 [25.5-31]	28 [25.3-31]	27.2 [24-30]	0.721
Blood pressure (mmHg)				
Systolic	111[103-122.5]	112.5 [104.5-121]	116 [105-121]	0.689
Diastolic	70 [64.5-76]	71 [65.5-76]	72.5 [69-78]	0.176
Glucose (mg/dl)	90 [84-97]	90 [84-96]	85 [82-95]	0.465
Total cholesterol (mg/dl)	190 [166-210]	189.3 [169-211]	190 [182-211]	0.378
HDL-C (mg/dl)	45 [36.4-55.7]	44.1 [35.6-53]	46 [38-50]	0.319
LDL-C (mg/dl)	114.5 [95.2-132]	117 [96-136]	119 [109-138]	0.227
Triglycerides (mg/dl)	142 [108-202]	157 [105-200]	145 [103-214]	0.859
CYP7A1	*rs10107182 C/T*			
Genotypes	*TT (n=758)*	*TC (n=261)*	*CC (n=15)*	*p-value*
Parameters				
BMI (kg/m^2)	27.9 [25.4-30.7]	28 [25.3-31]	27.2 [24-30]	0.721
Blood pressure (mmHg)				
Systolic	112[103-123]	112.5 [104.5-121]	116 [105-121]	0.692
Diastolic	70 [64.5-76]	70 [65.5-76]	72.5 [69-78]	0.182
Glucose (mg/dl)	90 [84-98]	90 [84-96]	85 [82-95]	0.467
Total cholesterol (mg/dl)	190 [166-210]	189.6 [169-211]	190 [182-211]	0.380
HDL-C (mg/dl)	45 [36.4-55.7]	44.1 [35.7-53]	46 [38-50]	0.321
LDL-C (mg/dl)	114.7 [95.3-132]	117 [96-136]	119 [109-137]	0.300
Triglycerides (mg/dl)	141 [108-201]	157 [105-199]	145 [103-214]	0.859
CYP7A1	*rs10504255 G/A*			
Genotypes	*AA (n=772)*	*AG (n=254)*	*GG (n=17)*	*p-value*
Parameters				
BMI (kg/m^2)	27.9 [25.4-30.8]	28 [25-31]	27.0 [24-29]	0.537
Blood pressure (mmHg)				
Systolic	111[103-122]	112.5 [104.5-121]	116 [103-120]	0.831
Diastolic	70 [64.5-76]	70.5 [65.5-76]	72.5 [69-78]	0.154
Glucose (mg/dl)	90 [84-97]	90 [84-96]	85 [82-97]	0.509
Total cholesterol (mg/dl)	190 [166-211]	189 [169-211]	201 [186-213]	0.185
HDL-C (mg/dl)	45 [36.5-56]	44.2 [35-53]	46 [38-51]	0.359
LDL-C (mg/dl)	114.6 [95.3-133]	116 [96-136]	119 [110-142]	0.223
Triglycerides (mg/dl)	142 [108-201]	156 [104-203]	145 [106-216]	0.809
CYP7A1	*rs81922870 T/G*			
Genotypes	*GG (n=736)*	*GT (n=288)*	*TT (n=20)*	*p-value*
Parameters				
BMI (kg/m^2)	28 [25.5-31]	28 [25-31]	26.6 [24-31]	0.650
Blood pressure (mmHg)				
Systolic	111[103-123]	112.3 [103.5-121]	116.5 [106-123]	0.310

Diastolic	70 [65-76]	70.5 [65-76]	74 [69-80]	0.099
Glucose (mg/dl)	90 [84-98]	89 [83-96]	86 [83-96]	0.484
Total cholesterol (mg/dl)	189 [166-211]	189 [169-210]	195 [176-210]	0.499
HDL-C (mg/dl)	45 [36.6-55]	45 [35-54]	45.5 [38-50]	0.330
LDL-C (mg/dl)	114.5 [95-133]	116 [95-135]	119 [106-138]	0.285
Triglycerides (mg/dl)	142 [108-203]	148 [101-198]	160 [114-213]	0.758

BMI: Body mass index, HDL: High-density lipoprotein – cholesterol, LDL: Low density lipoprotein, *p* = *p-value**. Data are expressed as median and interquartile interval (25th–75th). Analyzed as mean ± SD. * ANOVA and F-test.
